# CTR1 Silencing Inhibits Angiogenesis by Limiting Copper Entry into Endothelial Cells

**DOI:** 10.1371/journal.pone.0071982

**Published:** 2013-09-09

**Authors:** Gomathy Narayanan, Bharathidevi S. R., Harish Vuyyuru, Bharathselvi Muthuvel, Sulochana Konerirajapuram Natrajan

**Affiliations:** 1 R. S. Mehta Jain Department of Biochemistry and Cell Biology, Vision Research Foundation, Chennai, India; 2 Birla Institute of Technology and Science, Pilani (Rajasthan), India; Massachusetts General Hospital/Harvard Medical School, United States of America

## Abstract

Increased levels of intracellular copper stimulate angiogenesis in human umbilical vein endothelial cells (HUVECs). Copper transporter 1 (CTR1) is a copper importer present in the cell membrane and plays a major role in copper transport. In this study, three siRNAs targeting CTR1 mRNA were designed and screened for gene silencing. HUVECs when exposed to 100 µM copper showed 3 fold increased proliferation, migration by 1.8 - fold and tube formation by 1.8 - fold. One of the designed CTR1 siRNA (si 1) at 10 nM concentration decreased proliferation by 2.5 - fold, migration by 4 - fold and tube formation by 2.8 - fold. Rabbit corneal packet assay also showed considerable decrease in matrigel induced blood vessel formation by si 1 when compared to untreated control. The designed si 1 when topically applied inhibited angiogenesis. This can be further developed for therapeutic application.

## Introduction

Angiogenesis is a multistep complex process which involves growth of new blood vessels from the existing vasculature. This normally occurs in the physiological processes viz reproduction, development, wound healing [1], and in pathological process of inflammation, tumour growth, and neovascularisation [2]. Angiogenesis is regulated by several angiogenic stimulating factors like VEGF, bFGF, TGF β, as well as inhibitors such as endostatin and angiostatin [3]. Whenever proangiogenic factors exceed inhibitors, the angiogenesis disease factor predominates, which is pathological [4]. Thus, a balance is required between proangiogenic and antiangiogenic factors. Towards this end, inhibitors like Bevacizumab targeting VEGF, sorafenib and sunitinib targeting tyrosine kinase receptors are employed for controlling angiogenesis [5]. These drugs are beneficial, but they do show side effects like hypertension, anemia, hypothyroidism, thrombus formation in arteries and veins, bleeding complication [6], [7], and certain patients do not respond to anti - VEGF treatment [8]. There is a compelling need for developing molecules that can be used for therapeutic applications to treat angiogenesis related diseases.

Copper (Cu) is a micronutrient involved in various physiological functions such as respiration, acting as a catalytic cofactor, and in detoxification, iron absorption, and elastin cross linking [9]. Interestingly, increased Cu activates many factors like VEGF, bFGF, angiogenin, prostaglandin, ceruloplasmin, SPARC, NFκB while decreasing Cu decreases their function [10]. From the liver, Cu is transported, where copper transporter 1(CTR1) imports Cu which is sequestered through other Cu chaperones like ATOX, copper chaperone of superoxide dismutase 1 (CCS) and delivered to various other proteins for their functions [11]. In cell culture experiments, Hu *et al* have shown that Cu increased endothelial cells proliferation [12]. Furthermore, Sen *et al* demonstrated that Cu induced HIF1α in human keratinocytes, activated VEGF mediated migration [13]. In 2009, Feng et al showed that Cu is required for activation of HIF-1 α, including synthesis, stabilization, and translocation from the cytosol to nucleus [14]. This function of Cu requires CCS, as silencing CCS mRNA blocks insulin-like growth factor I (IGF-1) induces HIF-1α binding to hypoxia responsive element (HRE)and thereby decreases VEGF expression [15]. Mac Auslan and Gole have shown that Cu induced intraocular vascularisation and increased fibronectin synthesis in rats [16]. ATN- 224 a Cu chelator has been shown to reduce Cu induced angiogenesis in nude mice [17] indicating, the vital role of Cu in modulating angiogenesis.

CTR1, a high affinity Cu transporter, and CTR2, a low affinity Cu transporter belong to the family of copper transporters found in humans. CTR1 is a 32 kDa, membrane protein consisting of 190 amino acids. It is ubiquitously expressed and its gene is located in chromosome 9q32. CTR1 protein consists of three transmembrane α-helices, and oligomerization is required for its function [18]. Three highly conserved amino acid sequences in human CTR1 were observed to exhibit methionine-rich motifs Met150 and Met154, that are involved in Cu binding (MxMxxM, MMMMxM, and GxxxG) [19], [20]. High levels of CTR1 are present in heart, kidney, muscle and brain in humans [21]. Cu chelation is one of the strategies employed for treating angiogenesis, and the role of CTR1 as a major Cu importer in regulating angiogenesis is still unexplored. Recent studies have used siRNA for therapeutic applications in various diseases [22], [23]. In this report we present the data from *in vitro* and *in vivo* studies on the effect of CTR1 silencing that inhibited angiogenesis by limiting copper entry into endothelial cells.

## Materials and Methods

### Ethics statement

All the protocols involving the collection and processing of human samples were strictly adhered to the tenets of Helsinki declarations, and were approved by the Institutional Review Board of Vision Research foundation (IRB – VRF) where the study was conducted. IRB-VRF is the review committee consisting of members who are, highly reputed physician, justice, scientists, women, statistician, epidemiologist, medical doctors from several branches, lawyers and legal advisers, industrialist, etc., in charge of examining the ethical issues in the VRF research proposals. Reference number -150-2009-P is the IRB-VRF approval number for this work. Written consents were obtained from mothers who volunteered to donate their umbilical cord for research. Animal research protocols were in accordance with NIH guidelines for responsible animal care and use. The study was approved by Sri Ramachandra Medical College – Innovis committee on animal care and use of laboratory animals where the study was conducted. Reference number IAEC/XXVIII/SRU/2 12/2012 is the approval number from this committee for this animal work.

### Isolation of HUVECs

Human umbilical cords were processed within 3 h using a modified method of Baudin et al for isolation of HUVECs [24]. Every cord was washed with PBS and incubated at 37°C with 10 µg of collagenase (Sigma, USA) for 20 min. Cells were collected in endothelial – cell growth medium (EGM –2 CC-3156 with supplements CC-4176, Lonza) centrifuged for 10 min at 1500 rpm and the pellet was seeded onto fibronectin (Sigma) coated 25 cm^2^ flask kept at 37°C in 5% CO_2_. HUVECs were used between 3^rd^ to 6^th^ passages.

### Design and transient transfection of siRNA

The siRNAs were designed using Ambion web tools, and the rules described by Reynolds et al for siRNA designing were followed (table 1) [25]. Single stranded antisense CTR1 siRNA – si 1, si 2, si 3 and scrambled siRNA (Ssi) (Merck, India) of varying concentrations were used for the experiments. Each siRNA was incubated with 4 µL of transfection reagent ICA-fectine (Eurogentech, Belgium) for 15 min and then transfected onto HUVECs.

**Table 1:** List of antisense siRNAs used in the study.

	Sequence 5′– 3′
si 1	AGAAGGUUGCAUGGUACUGUU
si 2	AUGAGCAUGAGGAAGUAGCUU
si 3	AUCCACUACCACUGCCUUCUU
Ssi	GUGAUUUACGGGCUAGAGUAU

### Preparation of Cu and Penicillamine

Copper chloride (Sigma) 1 mM stock was prepared in EBM with 1% FBS medium, filter sterilised and required concentrations were used for the following experiments. Penicillamine, a known Cu chelator was used as a positive control. Penicillamine (P) stock of 1 mM was prepared in PBS and specified concentrations were used in the cell culture experiments.

### Cytotoxicity assay

For the MTT (3-(4,5-Dimethylthiazol-2-yl)-2,5-diphenyltetrazolium bromide) assay [26], 5000 cells/well in 96 well plate were exposed to Cu concentration of 10 nM to1 mM, CTR1 – siRNAs (si 1, si 2, and si 3), Ssi and P 800 µM for 1 h. After exposure, cells were treated with MTT and the formazan crystals formed were dissolved in DMSO and read at 570 nm in spectra max fluorescent plate reader (Molecular devices, USA).

### Real time PCR

For the real time PCR experiments, cells were exposed to Cu 100 µM, siRNAs of concentration 1 nM and 10 nM each and 800 µM P in the presence of Cu for 1 h after starving the HUVECs in above mentioned EBM (endothelial cell basal medium+1% FBS) for 4 h. Total RNA was extracted from HUVECs using TRIzol reagent (Sigma) by following manufacture's protocol and was quantified using NanoDrop ND-1000 spectrophotometer. Total RNA 1 µg was used to synthesize cDNA (iScript cDNA synthesis kit, Biorad, USA) and 0.25 µl of cDNA was used to study the mRNA expression of CTR1. Quantitative real-time PCRs [27] were performed using Applied Biosystems 7300 with SYBR Green chemistry (Eurogentec, Belgium). Primers for qRT-PCR (table 2) and RT-PCR (table S1) for CTR1, GAPDH and VEGF were commercially purchased (Eurogentec, Belgium). Real-time PCR cycle conditions included the following steps: denaturation at 95°C for 2 min, followed by 40 cycles of denaturation at 95°C for 10 sec, annealing at 60°C for 20 sec and extension at 72°C for 25 sec. Each sample was run in triplicate and *C_t_* was determined for the target transcripts.

**Table 2:** List of primers used in this study.

S.No	Gene Name	Accession No.	Forward Primer	Reverse Primer
1.	CTR1	NM_001859.3	5′-GCG TAA GTC ACA AGT CAG CAT TC- 3′	5′-GCG TAA GTC ACA AGT CAG CAT TC- 3′
2.	VEGF 165	NM_0003376.5	5′-CGG TAT AAG TCC TGG AGC GTT C-3′	5′- GCC TCG GCT TGT CAC ATC TG- 3′
3.	GAPDH	NM_002046	5′-GAA CAT CAT CCC TGC CTC TAC TG- 3′	5′-CGC CTG CTT CAC CAC CTT C- 3′

### Immuno cytochemistry, ELISA, and western blot

HUVECs were starved in 1% EBM for 4 h and exposed to Cu 100 µM, Cu 100 µM+si 1- 10 nM, Cu 100 µM+Ssi 10 nM and Cu 100 µM+P 800 µM for 6 h, cells without Cu were taken as control. Cells were doped with 10 nM siRNA for every 2 h for a period of 6 h.

For ICC, HUVECs were grown on chamber slides and then exposed to the above mentioned conditions. Cells were fixed in 4% paraformaldehyde (Merck, India) for 20 min and permeabilized for 10 min with 0.1% triton ×100 (Sigma). Blocking was done with 3% BSA with 0.1% triton ×100 for 30 min at room temperature. Slides were incubated with 1∶50 anti- human CTR1 raised in rabbit (Santa Cruz) overnight at 4°C and 1∶500 anti - rabbit secondary antibody (Santa Cruz) for 2 h and developed with 3, 3′-diaminobenzidine (DAB).

For CTR1 ELISA (USCN, USA) cells were grown in six well plates and exposed to conditions mentioned earlier under ICC and lysed in M-PER (mammalian protein extraction reagent, Pierce, USA).Cell lysates were centrifuged at 1500 rpm for 10 min, 50 µL of the supernatants were used for ELISA.

For Western blot, proteins (Bradford assay kit, Pierce, USA) 50 µg were separated in 10% SDS gel and transferred to nitrocellulose membranes. Membranes were blocked with 5% milk PBST for 1 h and incubated with CTR1 antibody (1∶100), anti-human β-actin raised in mouse (1∶500), anti-human VEGF raised in rabbit (1∶200) (Santa Cruz, USA) overnight at 4°C. The membranes were washed thrice with PBST, and incubated with (1∶7500) diluted anti - rabbit HRP and anti - mouse (1∶5000) HRP respectively for 2 h (Santa Cruz, USA). The membranes developed using ECL PLUS reagent (GE healthcare, USA) and images were documented using Flurochem CS3 (Bioscreen, India).

### Intracellular copper estimation

#### Phen Green staining

For intracellular Cu estimation cells were grown in six well plates, starved for 4 h in 1% EBM. Cells were exposed to Cu 100 µM, Cu 100 µM+si 1- 10 nM, Cu 100 µM+Ssi 10 nM and Cu 100 µM+P 800 µM for 1 h whereas cells not exposed to Cu were taken as control. Trypsinized cells were stained with 5 µM *Phen Green FL* dye (Invitrogen, USA) and incubated for 30 min at 37°C. Cells were washed and resuspended in PBS, 1×10^4^ cells were gated and analyzed by FACS calibur (BD, USA). Intracellular Cu binds to phen Green dye and decreases the fluorescence which was measured as median fluorescent index at excitation/emission – 490 nm/528 nm [28]. The extent of decrease in fluorescence is the index of Cu entry, and vice versa.

#### Atomic absorption spectroscopy

Intracellular Cu levels were also measured by Atomic absorption spectroscopy (AAS) AAnalyst 700 (Perkin Elmer, USA). Cells were grown in 25 cm^2^ flask, starved with 1% EBM for 4 h. HUVECs were exposed to Cu 100 µM, Cu 100 µM+si 1 - 10 nM, Cu 100 µM+Ssi 10 nM, Cu 100 µM+P 800 µM for 1 h and cells without Cu were taken as control. Cells were washed with PBS and lysed in (5∶1) nitric acid: perchloric acid (Merck, India) and then ashed. Samples were homogenized in 200 µL of 0.2% nitric acid, centrifuged, and the supernatant was taken for Cu estimation by AAS. Standard Cu (1 mg/ml, Perkin Elmer) was used for calibration. Cu was estimated at 324.8 nm using a hallow cathode lamp. The slit was maintained at 0.7 nm, Cu was atomized at 2300°C using graphite furnace system and detected by spectrophotometer.

### *In vitro* angiogenesis assay

#### Proliferation assay

To examine the effect of Cu and CTR1 siRNA on cell proliferation, cells were seeded at the density of 3000 cells/well in 0.1% gelatin coated 24 well plates. HUVECs were grown in EGM - 2 medium till 60% confluency and then exposed to Cu 100 µM, Cu 100 µM+si 1 - 10 nM, Cu 100 µM+Ssi 10 nM and Cu 100 µM+P 800 µM in 5% EBM in the presence of 100 nCi tritiated thymidine for 48 h whereas cells without Cu were taken as control. Cells were lysed in 1% SDS with 0.1 N NaOH and heated in 60°C for 1 h, and then 100 µL of the lysates was taken for analysis. The amount of radioactivity was measured in disintegrations per min (DPM) using liquid scintillation counter LS6500 (Beckman coulter, USA).

### Apoptosis assay

HUVECs were grown in six well plates, starved with 1% EBM for 4 h. Then, exposed to Cu 100 µM, Cu 100 µM+si 1 - 10 nM, Cu 100 µM+Ssi 10 nM, Cu 100 µM+P 800 µM for 24 h and cells without Cu were taken as control. Cell lysates were taken for the apoptosis assay using cell death ELISA kit (Roche, Swiss) by following manufacturer's protocol.

### Tube formation assay

For the tube formation, assay cells were first transfected with CTR1 siRNA in the presence of Cu 100 µM and Cu+P 800 µM and cells without Cu were taken as control. Cells were seeded on to the matrigel (Chemicon, USA) and observed for tube formation till 8 h at 37°C in 5% CO_2_. Cells were fixed in 4% paraformaldehyde and photographs were taken using phase contrast microscope Axio observer (Ziess, USA). Five randomly selected fields were photographed in each well, and the total number of junctions was analyzed using angioquant software v1.33.

### Attachment assay

Cell attachment assay was performed in 12 well plates coated with 0.1% gelatin. Cells were exposed to the above mentioned condition for 30 min in a vial and then allowed to attach on the plate for 30 min. Unattached cells were washed with PBS. Attached cells were fixed with 4% paraformaldehyde stained with Giemsa for 15 min, photographed and counted using image j software NIH.

### Migration assays

#### Wound healing assay

Cells were grown in six well plates to confluency and then starved for 4 h in 1% EBM medium. A scratch was created with a microtip, and the cells were exposed to same as before mentioned experimental conditions. Cells were observed for migration under phase contrast microscope (Nikon, Japan) and documented [29].

#### Transwell migration assay

For quantitative assessment of migration, 2×10^4^ cells were plated on a transwell membrane of PET 8-µm pore size onto the upper chamber (Millipore, Switzerland) coated with 0.1% gelatin and cells were exposed to the above mentioned conditions for 16 h. Non migrated cells on the upper side of the filter were removed with a cotton swab, and the migrated cells were fixed with 4% paraformaldehyde in PBS and stained with Giemsa stain washed and dried. The migrated cells were photographed. Relative cell migration was determined by counting the number of the cells migrated in control versus treated condition using image j software NIH [30].

### *In vivo* angiogenesis assay

#### Rabbit corneal packet assay

Two animals were taken in each group. Group 1– matrigel implanted and PBS treated were used as vehicle controls and Group 2– matrigel implanted si 1 100 ng with 2′ – O – methyl modification treated were used in the study. All the animals in the study were operated in the right eye to implant the matrigel (BD, USA), whereas the left eye was taken as untreated control. New Zealand white male rabbits (2 kg) were subcutaneously injected with ketamine (50 mg/kg) and xylazine (5 mg/kg) to anesthetize. Using a central intrastromal linear keratomy (∼2.5 mm in length) was made with a surgical knife. A lamellar micro pocket was dissected to 2 mm near the limbus and 50 µL of matrigel (BD) was implanted. Antibiotic ointment (erythromycin) was applied once to the surgical eye to prevent infection and to decrease irritation of the irregular ocular surface. On postoperative days 3, 5, 7, 9, and 11 after gel implantation 100 ng of si 1 was topically dispensed in the form of drops twice a day. At the end of 11^th^ day, the animals were photographed using stereomicroscope SMZ1000 (Nikon, Japan) and euthanized.

### Statistical analysis

All the experiments were done in triplicates. Data are expressed as mean ± SD. Differences between the means of unpaired samples were evaluated by Student's *t* test and *p* values<0.05 were considered to be statistically significant.

## Results

### Cytotoxicity assay

**Figure 1:**
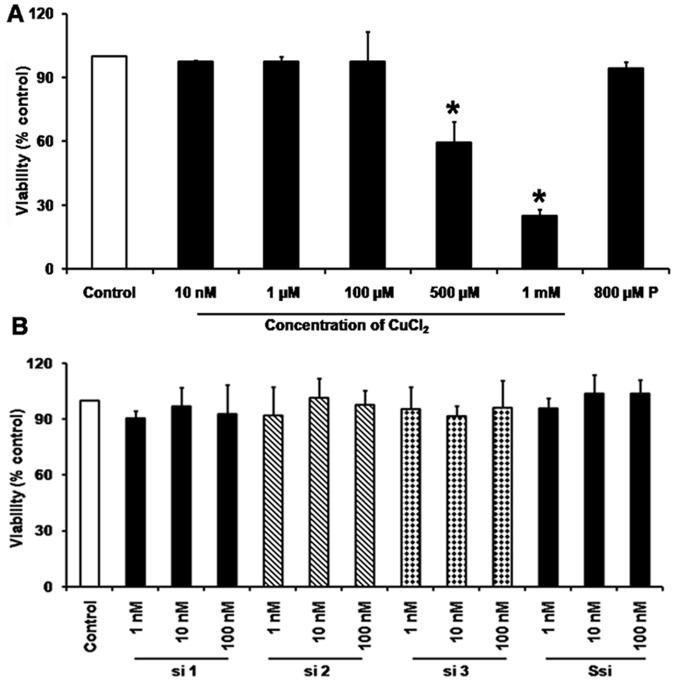
Cytotoxicity assay. A. MTT assay for varying Cu concentrations: Cu concentration of 10 nM–1 mM in HUVECs cells showed that the IC_50_ for Cu to be 500 µM. B. MTT assay for different siRNAs: Cytotoxic effect of different siRNAs concentration from 1 nM–100 nM were tested and found to have no cytotoxic effect.

To test the cytotoxic effect of Cu on HUVECs, MTT assay was done by incubating the cells with varying concentrations Cu from 10 nM–1 mM. The IC_50_ was found to be 500 µM for Cu (Fig. 1A). To test for the cytotoxicity of siRNA on HUVECs, three different siRNAs si 1, si 2, si 3 (1 nM–100 nM) targeting CTR1 and Ssi (1 nM–100 nM) without Cu was tested and found to be non cytotoxic (Fig. 1B).

### Optimisation of Cu and Penicillamine concentration

HUVECs were exposed to Cu in concentrations ranging from 1 µM to 300 µM for 1 h. CTR1 showed a dose dependent increase in mRNA expression when exposed to Cu. CTR1 expression was high in Cu 100 µM concentration and higher concentrations did not show any significant change (Fig. S1A). Similarly, Penicillamine (P) a copper chelator was used as control in all the experiments. Concentrations of 400 µM and 800 µM were exposed to cells in the presence of Cu 100 µM. P 800 µM concentration was found to be effective in reducing the Cu induced CTR1 mRNA expression in HUVECs (Fig. S1B). All data were normalised to GAPDH expression.

### CTR1 siRNA inhibits CTR1 mRNA and protein expression

**Figure 2:**
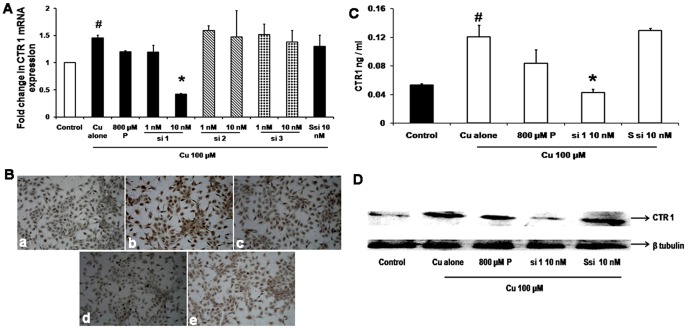
CTR1 silencing inhibits mRNA and protein expression. A. Real time PCR for CTR1: qRT-PCR shows that Cu 100 µM treatment showed an increase in 45% CTR1 mRNA expression (# *p* = 0.01) compared to cells without Cu. CTR1 si 1 at 10 nM concentration significantly (* *p* = 0.01) decreased the CTR1mRNA expression by 71% when compared to 100 µM Cu whereas si 2, si 3 at 10 nM showed no inhibition. P 800 µM showed 14% decreased in expression of CTR1 when compared to Cu treated. B. Immuno cytochemistry for CTR1: a - Control, b - Cu 100 µM, c- Cu 100 µM+P 800 µM, d - Cu 100 µM+si 110 nM. CTR1 protein staining showed an increase in staining intensity with Cu treatment, (shown as brown colour) and decrease in staining intensity with si 110 nM treatment. C. CTR1 ELISA: HUVECs were treated with Cu 100 µM showed a significant (# *p* = 0.05) increase in CTR1 protein level compared to control and si 1 10 nM showed a significant decrease (* *p* = 0.04) in protein level when compared to Cu. D. Western blot for CTR1: Immunoblot showed an inhibition of CTR1 protein with si 110 nM treated condition compared to Cu which was not observed in 800 µM P treatment.

qRT-PCR of 100 µM Cu treatment showed an increase by 45% in CTR1 mRNA expression (*p* = 0.01) compared with control. CTR1 mRNA silencing effect of all the three siRNAs in the presence of Cu 100 µM showed that 10 nM concentration of si 1 decreased CTR1 expression by 71%, whereas si 2 and si 3 did not show any significant effect. Similarly Ssi 10 nM and P 800 µM also did not show any significant decrease in mRNA expression when compared with Cu treated cells. GAPDH was used as the house keeping gene (Fig. 2A) to normalise CTR1 expression. Since si 1 showed a significant decrease in CTR1 expression it was chosen for further experiments.

Immunocytochemistry by DAB showed an increase in CTR1 protein in the presence of 100 µM Cu and si 1 - 10 nM decreased protein expression. Similarly, P 800 µM treated showed a mild decrease when compared to Cu treated (Fig. 2B) whereas Ssi 10 nM did not show any significant change.

To confirm this, CTR1 ELISA was performed which showed that cells treated with Cu 100 µM had a significant (*p*<0.05) increase in CTR1 protein level (0.12±0.022 ng/mL) compared with control (0.05±0.003 ng/mL) and the si 1 - 10 nM showed a significant decrease *p* = 0.04 (0.04±0.006 ng/mL) when compared to Cu treated. P 800 µM and Ssi 10 nM treated did not show any significant change when compared to Cu treated (Fig. 2C).

To validate further, Western blot for CTR1 was done, and it showed an increase in CTR1 protein level in the presence of Cu, and decreased when treated with si 1 - 10 nM. While, P 800 µM treated also did not show any significant change in signal intensity when compared to Cu treated (Fig. 2D).

### CTR1 si RNA inhibits copper entry

**Figure 3:**
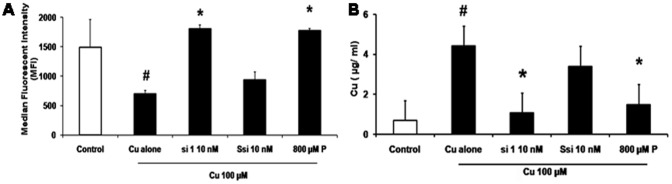
CTR1 silencing inhibits Cu entry. A. Cu estimation by FACS: Histogram for Phen Green analysis was plotted with the mean fluorescent intensity obtained. Cu estimation by FACS, showed that treatment with Cu (# *p* = 0.04) increased the intracellular Cu levels (indicated by a decrease fluorescent intensity) and si 1 - 10 nM (*p = 0.02) decreased intracellular Cu levels in the presence of Cu, (indicated by an increase in fluorescent intensity). Ssi showed no significant effect and P 800 µM also showed significant inhibition (*p = 0.03) of Cu inside the cell. B. Cu estimation by AAS: Intracellular Cu levels increased significantly with Cu100 µM treatment (# *p* = 0.0005) and si 1 - 10 nM treatment decreased Cu levels (* *p* = 0.0008), even though P 800 µM also showed (* *p* = 0.01), a decrease in the Cu level statistically significant.

Since copper increased CTR1 expression, it was imperative to know the intracellular Cu levels. Phen Green dye binds to Cu and reduces fluorescence. Cu estimation by FACS using Phen Green dye showed that 100 µM Cu reduced the median fluorescence intensity by 50% which was significant (*p* = 0.04) when compared with control cells without Cu. Cu 100 µM+si 1 - 10 nM showed an increase in median fluorescence intensity by 60% which was significant (*p* = 0.02) when compared to cells treated with Cu. Similarly, P 800 µM treated also showed 60% which was significant (*p* = 0.03) increase in fluorescence intensity when compared to Cu treated (Fig. 3A).

To reaffirm, Cu estimation was done by AAS method in cell lysates. Cells exposed to Cu 100 µM (4.42 µg/mL) showed a 6.4 fold increase in Cu levels when compared with cells without Cu treatment (0.69 µg/mL), (*p* = 0.0005). Cells treated with si 1 - 10 nM showed a fourfold decrease in Cu levels (1.08 µg/mL) significantly (*p* = 0.0008) when compared with Cu. P 800 µM also showed a 2.9 - fold decrease in the Cu level (*p* = 0.01), which also showed statistical significance. No significant changes in Cu levels were seen in the presence of Ssi (Fig. 3B).

### CTR1 siRNA inhibits angiogenesis

**Figure 4:**
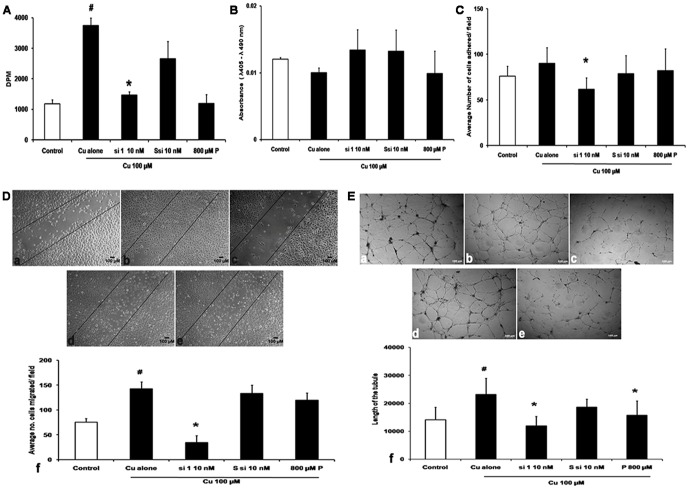
CTR1 silencing inhibits angiogenesis. A. Proliferation assay: Triated thymidine was used for proliferation assay. Cu treatment showed an increase in proliferation (# *p* = 0.0003) and si 1 - 10 nM showed inhibition of proliferation (* *p* = 0.003), Ssi at 10 nM concentration did not show any effect, whereas Cu+P also significantly inhibited proliferation (* *p* = 0.02). B. Apoptosis assay: Apoptosis assay by Cell death ELISA kit showed no apoptosis in all conditions tested. C. Tube formation assay: a - Control, b- Cu 100 µM, c- Cu 100 µM+si 1 - 10 nM, d- Cu 100 µM+Ssi 10 nM, e- Cu 100 µM+P 800 µM. Cu 100 µM increased tube formation seen in the length of the tubule in Cu treated when compared with cells without Cu. Cu+si 1 - 10 nM reduced tube formation by decreasing the length of the tubule (*p = 0.01), when compared to Cu treated, which was not observed in Cu 100 µM+Ssi 10 nM. P 800 µM treatment decrease in length of the tubule (*p = 0.01) when compared to Cu. g- Histogram representing the length of the tubule in various conditions. D. Migration assay: Scratch assay a -Control, b- Cu 100 µM, c- Cu 100 µM+si 1 - 10 nM, d- Cu 100 µM+Ssi 10 nM, e- Cu 100 µM+P 800 µM (Magnification - 10×). HUVECs treated with Cu 100 µM increased the cell migration whereas si 1 - 10 nM inhibited migration, and no inhibition was in the presence of Ssi 10 nM and P 800 µM treatment. f- Transwell migration assay: Histogram showed that Cu 100 µM increased the cell migration significantly (# *p* = 0.00001), and Cu+si 1 - 10 nM reduced migration significantly (* *p* = 0.00001), Ssi 10 nM showed no effect, whereas P 800 µM treated did not inhibit migration. E. Attachment assay: a -Control, b- Cu 100 µM, c- Cu 100 µM+si 1 - 10 nM, d- Cu 100 µM+Ssi 10 nM, e- Cu 100 µM+P 800 µM HUVECs, f- histogram of cells counted in average of five fields. Cells showed that Cu 100 µM increased the cell attachment to the gelatin matrix significantly (# *p* = 0.02) and Cu+si 1 - 10 nM treatment reduced the number of cells attaching (* *p* = 0.00009), Ssi 10 nM showed no effect; whereas P 800 µM treated showed reduction in the number of cells attached which not significant.

Cell proliferation assay showed that Cu 100 µM induced proliferation of the HUVECs cells by threefold (*p* = 0.0003) compared to cells without Cu treatment, and 2.5 fold decrease in proliferation was seen in Cu 100 µM+si 1 - 10 nM (*p* = 0.003) compared to Cu treated, Cu 100 µM+Ssi 10 nM did not show any significant decrease in proliferation whereas Cu 100 µM+P 800 µM showed a decrease of 2.8 fold (*p* = 0.02) when compared with Cu (Fig. 4A).

Apoptosis assay indicated that there was no cell death induced by siRNA as verified by cell death ELISA kit. We measured the histone DNA complexes in cytoplasm an early apoptosis marker and the decrease in proliferation may be due to other mechanism (Fig. 4B).

Attachment assay with Cu 100 µM showed more attachment of cells to gelatin matrix as compared to control cells with a *p* value of 0.02 and Cu 100 µM+si 1 - 10 nM decreased attachment of HUVECs with a *p* value of 0.00009 respectively. Cells exposed to Cu 100 µM+Ssi 10 nM showed no effect whereas Cu 100 µM+P 800 µM treated cells showed a reduction in the number of cells attached which was not significant (Fig. 4C).

In wound healing assay, Cu 100 µM showed increased migration of cells and Cu 100 µM+si 1 - 10 nM concentration inhibited the migration. Quantification of migration was done using transwell inserts 8 µM size where Cu showed 88% increase in migration of cells with a *p* value of 0.00001 when compared to control cells and si 1 - 10 nM concentration reduced migration by 75% with a *p* value of 0.00001 when compared to Cu 100 µM treatment, whereas Cu 100 µM+Ssi 10 nM and Cu 100 µM+P 800 µM treated cells did not inhibit migration (Fig. 4D).

Tube formation assay showed that Cu 100 µM increased tube formation indicated by increase in tubule length by 1.8 fold (*p* = 0.04) in Cu 100 µM, when compared with cells not treated with Cu. Treatment with Cu 100 µM+si 1 - 10 nM concentration showed 2.8 - fold decrease in tube length (*p* = 0.01) when compared to cells treated with Cu. Cu 100 µM+Ssi 10 nM did not show any significant change. Cu 100 µM+P 800 µM treated condition showed 2.5 fold decrease in tube length (*p* = 0.01), when compared cells only treated with Cu. Quantification was done using angioquant software in which the length of the tubule was measured, and it was found that Cu increased the tube formation and siRNA and P 800 µM decreased the same (Fig. 4E).

**Figure 5:**
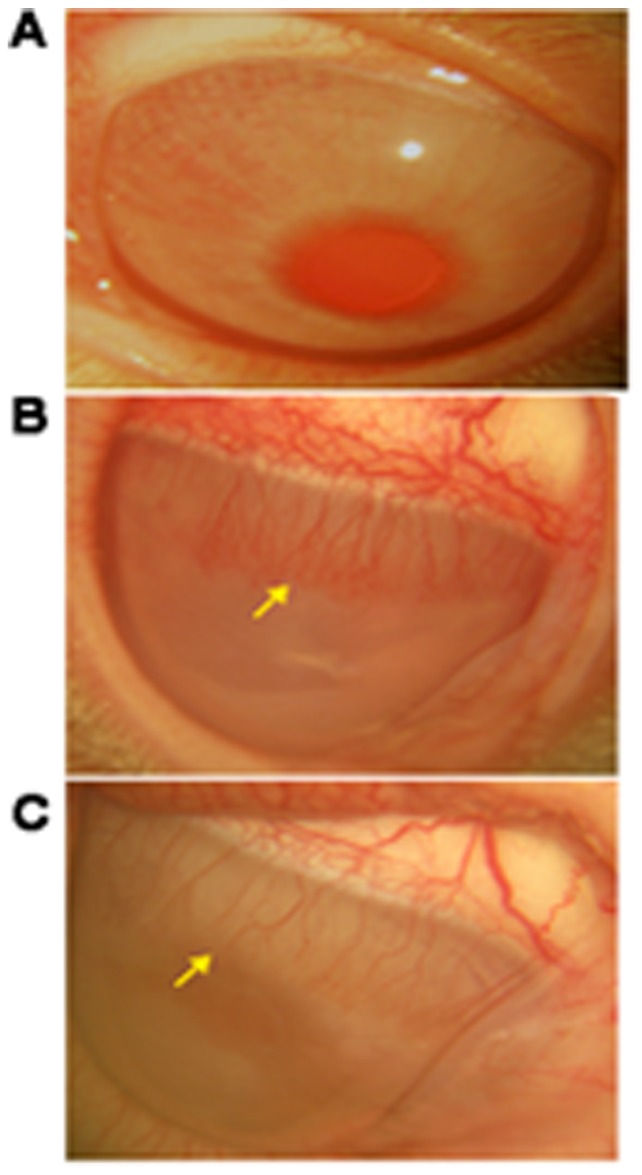
CTR1 siRNA inhibited angiogenesis. A. Untreated eye. B. Vehicle control- showed matrigel induced microvascular blood vessel formation (indicated in arrow). C. si RNA (si 1 100 ng) treated – showed decreased blood vessel formation after treatment with siRNA (indicated in arrow).

CTR1 siRNA (si 1 100 ng), which was 2′– O – methyl modified for better stability was tested *in vivo* by performing corneal packet assay which showed regressed blood vessel formation when compared to matrigel treated animal. The regression of blood vessels was seen from day 3, but we waited till day 11 where the results were significant. PBS given as a vehicle control did not show regression in blood vessel formation when compared with matrigel implant (Fig. 5).

### CTR1 siRNA inhibits angiogenesis by decreasing VEGF 165 levels

**Figure 6:**
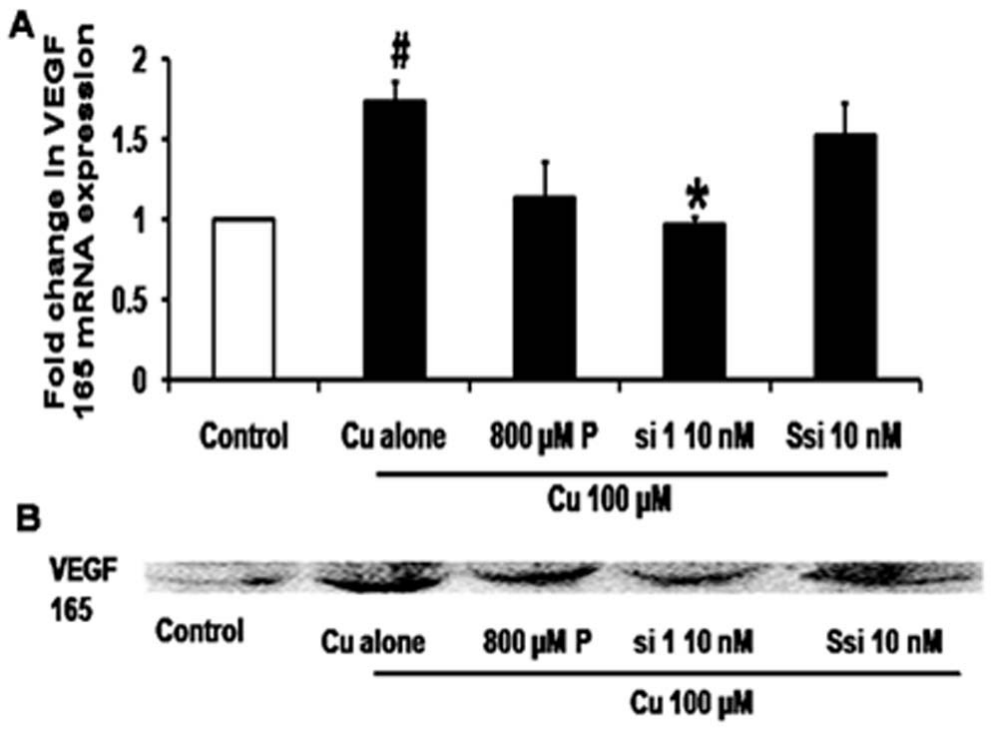
CTR 1 silencing inhibits VEGF 165. A.VEGF real time PCR: qRT PCR shows that Cu 100 µM treatment showed a 1.73 fold increase in VEGF165 mRNA expression (#*p* = 0.013) compared to control. CTR1 si 1 at 10 nM concentration decreased the VEGF expression by 50% when compared to Cu (*p = 0.015), P 800 µM showed a mild decrease when compared with Cu, whereas Ssi 10 nM did not show any significant change B. Western blot: Cu increased VEGF protein levels, si 1 - 10 nM decreased of VEGF 165 protein when compared with Cu 100 µM treatment. P 800 µM showed slight reduction in protein level whereas Ssi 10 nM did not show any significant change when compared to Cu treatment.

Cu has been shown to induce VEGF expression through a similar pathway to HIF 1α pathway, and it has also been showed that it was inhibited by Cu chelating agents [13]. In this study we observed that Cu 100 µM increased VEGF mRNA levels by 1.73 - fold (*p* = 0.015) when compared control cells and reduced in the presence of si 1 - 10 nM by 45% (*p* = 0.013) when compared to cells treated with Cu. P 800 µM treatement showed decreased mRNA levels by 1.12 - fold whereas Ssi showed no significant change. Protein expression showed increased VEGF protein in the presence of Cu and inhibiting Cu entry inside the cell by silencing the CTR1 decreased VEGF expression (Fig. 6A and B).

## Discussion

Copper from the liver is transported through ceruloplasmin to the cells. CTR1 transports the Cu (I) across the plasma membrane. The Cu which enters the cell attaches to copper chaperones COX17, andCCS which delivers the Cu to COX and SOD, as well as glutathione and metallothioneins [31], [32], [33]. The levels of intracellular Cu are regulated not only by the Cu importer CTR1 but also by the Cu exporter ATP7A (Menkes ATPase), whose function is achieved through copper-dependent translocation from trans-golgi network [34], [35].

Cu is involved in modulating angiogenesis by altering the growth factors and also the extracellular matrix. Increased copper levels are reported in various tumors where Cu levels are observed to increasewith disease progression. Goodman et al suggested that Cu deficiency can act as an anti cancer strategy [18].

Davis et al showed for the first time that systemic administration of siRNA targeting M2 subunit of ribonucleotide reductase protein in malignant melanoma can be beneficial [36]. In ocular diseases like neovascular AMD where angiogenesis is involved, Kaiser et al has demonstrated that a single intra vitreal dose of siRNA (1600 µg/eye) targeting VEGF R1 improves visual acuity [37]. RNAi technology is effective in mammalian cells. Work done by Javier Martinez et al, 2002 showed that single stranded antisense si RNA can lead to targeted cleavage of mRNA; by using 5′ phosphorylated single siRNA which silenced nuclear envelope protein effectively in Hela cell lines [38]. Later Torgier Holen et al showed that both the double stranded and single stranded si RNA share the common pathway of inhibiting the target gene where human tissue factor mRNA was targeted [39]. There is a recent study in which the copper chaperone for Cu, Zn- SOD (CCS), has been silenced with siRNA to elucidate the role of Cu in cobalt induced HIF 1 alpha expression. It was found that Cu is required even for the transcription activation of VEGF induced by HIF1α [40].

In this study, CTR1 mRNA was targeted by siRNA. The treatment did not affect the viability of the cell for all the concentrations tested. The IC 50 for Cu was 500 µM (Fig. 1A) and Cu 100 µM was sufficient to induce proliferation of HUVECs.

CTR1 protein levels increased in the presence of Cu. The inhibition of CTR1 by si 1 - 10 nM reduced the Cu levels, CTR1 protein and the CTR1 mRNA expression by 50% (Fig. 3). Addition of Cu increased proliferation, migration and tube formation of HUVECs, the siRNA treatment along with Cu inhibited the Cu induced effect leading to reduced proliferation, migration and tube formation (Fig. 4). *In vivo* study showed that the same siRNA (100 ng) showed a regression in blood vessel formation when compared to matrigel treated control (Fig. 5).

In cell culture experiments, si RNA treatment showed a better inhibition than the standard penicillamine treatment which is a control in this study. Cu chelators like penicillamine and tetrathiomolybdate are widely employed to reduce Cu levels in tumours [41]. Brem *et al* showed in clinical trials that penicillamine at a concentration of 1.6 m mol/day to a maximum of 19 m mol/day along with radiation therapy was used to treat patients with glioblastoma [42]. In our study, we could see the effect of penicillamine only at 800 µM concentrations whereas CTR1 si RNA showed good effect in 10 nM concentration, the scrambled siRNA did not show any of the effects confirming the specific role of CTR1 siRNA.

To verify the mechanism involved in the inhibition of angiogenesis by CTR1 the VEGF expression was measured in the presence of Cu with and without siRNA and it showed that Cu 100 µM increased VEGF mRNA expression by 1.5 fold by real time PCR and also there was an increase in the protein expression by Western blot (Fig. 6). Chandan et al studied Cu induced VEGF expression, thereby leading to wound closure in HaCaT cell lines [13]. This is the first study to show that the intracellular Cu levels can be reduced by targeting CTR1. The designed CTR1 siRNA not only reduced the Cu levels but also inhibited Cu induced angiogenesis. Copper exporter ATP7A levels were seen to be unaltered in the presence of si 1 (data not shown). Other Cu chaperones and Cu enzymes have not been studied in the presence of si 1 in this study. This siRNA can be conjugated with tissue specific peptides for targeted delivery. Further this molecule is being studied for its stability and bioavailability to develop it as a therapeutic molecule for the treatment of diseases associated with abnormal angiogenesis.

## Supporting Information

Figure S1 **Varying Cu and Penicillamine concentration.** A. RT PCR for CTR1with varying Cu concentration: lane 1 – control, lane 2- 1 µM Cu, lane 3- 10 µM Cu, lane 4 – 100 µM Cu, lane 5 – 200 µM Cu, lane 6 - 300 µM Cu, lane 7 molecular weight ladder. PCR showed increased CTR1 expression in 100 µM Cu with a product size of 237 bp length. All the samples were normalised to GAPDH which had a product size of 495 bp. B. RT PCR for CTR1 with varying P concentration: lane 1 – molecular weight ladder, lane 2-control, lane 3- 1 µM Cu, lane 4- 100 µM Cu, lane 5 – 100 µM Cu+400 µM P, lane 6 – 100 µM Cu+800 µM. PCR showed decreased CTR1 expression in a dose dependent manner in the presence of 400 µM P and 800 µM P in the presence of Cu with a product size of 237 bp length. All the samples were normalised to GAPDH which had a product size of 495 bp.
(DOC)

Table S1 **Primer sequence used for RT – PCR.**
(DOC)
