# Supplementary material for: CTR1 Silencing Inhibits Angiogenesis by Limiting Copper Entry into Endothelial Cells
Source: PLoS One. 2013 Sep 9;8(9):e71982. doi: 10.1371/journal.pone.0071982 (PMC3767743; doi:10.1371/journal.pone.0071982)
Supplement: Figure S1 — Varying Cu and Penicillamine concentration. A. RT PCR for CTR1with varying Cu concentration: lane 1 – control, lane 2- 1 µM Cu, lane 3- 10 µM Cu, lane 4 – 100 µM Cu, lane 5 – 200 µM Cu, lane 6 - 300 µM Cu, lane 7 molecular weight ladder. PCR showed increased CTR1 expression in 100 µM Cu with a product size of 237 bp length. All the samples were normalised to GAPDH which had a product size of 495 bp. B. RT PCR for CTR1 with varying P concentration: lane 1 – molecular weight ladder, lane 2-control, lane 3- 1 µM Cu, lane 4- 100 µM Cu, lane 5 – 100 µM Cu+400 µM P, lane 6 – 100 µM Cu+800 µM. PCR showed decreased CTR1 expression in a dose dependent manner in the presence of 400 µM P and 800 µM P in the presence of Cu with a product size of 237 bp length. All the samples were normalised to GAPDH which had a product size of 495 bp. (DOC) [file pone.0071982.s001.doc]

**Figure S1**


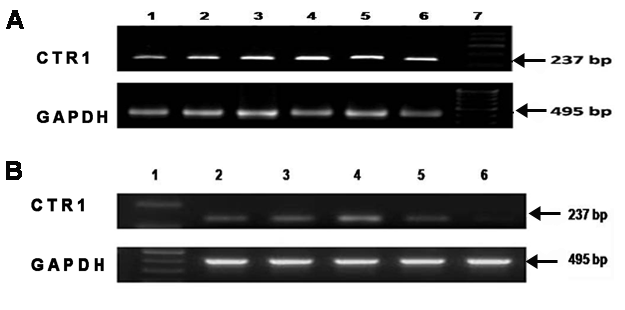


**Figure S1: Varying Cu and Penicillamine concentration**

A. RT PCR for CTR1with varying Cu concentration : lane 1 – control, lane 2- 1 µM Cu, lane 3- 10 µM Cu, lane 4 – 100 µM Cu, lane 5 – 200 µM Cu, lane 6 - 300 µM Cu, lane 7 molecular weight ladder. PCR showed increased CTR1 expression in 100 µM Cu with a product size of 237 bp length. All the samples were normalised to GAPDH which had a product size of 495 bp.

B. RT PCR for CTR1 with varying P concentration: lane 1 – molecular weight ladder, lane 2-control, lane 3- 1 µM Cu, lane 4- 100 µM Cu, lane 5 – 100 µM Cu + 400 µM P, lane 6 – 100 µM Cu + 800 µM. PCR showed decreased CTR1 expression in a dose dependent manner in the presence of 400 µM P and 800 µM P in the presence of Cu with a product size of 237 bp length. All the samples were normalised to GAPDH which had a product size of 495 bp.
