# Supplementary material for: CTR1 Silencing Inhibits Angiogenesis by Limiting Copper Entry into Endothelial Cells
Source: PLoS One. 2013 Sep 9;8(9):e71982. doi: 10.1371/journal.pone.0071982 (PMC3767743; doi:10.1371/journal.pone.0071982)
Supplement: Table S1 — Primer sequence used for RT – PCR. (DOC) [file pone.0071982.s002.doc]

Table S1: Primer sequence used for RT - PCR

| TARGET GENE | FORWARD PRIMER | REVERSE PRIMER |
| --- | --- | --- |
| CTR1 | 5’ CTTAGACTGGCTGCCAAAGG 3’ | 5’AGAGTAAGGGGGCCAAAGAA 3’ |
| GAPDH | 5’ GCCAAGGTCATCCATGACAAC 3’ | 5’GTCCACCACCCTGTTGCTGTA 3’ |
